# Multiple Soft Tissue Sarcomas in a Single Patient: An International Multicentre Review

**DOI:** 10.1155/2018/5392785

**Published:** 2018-04-01

**Authors:** Johnathan R. Lex, Ahmed Aoude, Jonathan D. Stevenson, Jay S. Wunder, Scott Evans, Peter C. Ferguson, Nikolaos A. Stavropoulos, Lee Jeys, Krista Goulding, Robert E. Turcotte

**Affiliations:** ^1^University of Birmingham, Birmingham B15 2TT, UK; ^2^McGill University Health Centre, Montreal, QC, Canada H4A 3J1; ^3^Royal Orthopaedic Hospital, Birmingham B31 2AP, UK; ^4^University of Toronto Musculoskeletal Oncology Unit, Mount Sinai Hospital, Toronto, ON, Canada M5G 1X5; ^5^School of Life and Health Sciences, Aston University, Birmingham B4 7ET, UK

## Abstract

Developing multiple soft tissue sarcomas (STSs) is a rare process, sparsely reported in the literature to date. Little is known about the pattern of disease development or outcomes in these patients. Patients were identified from three tertiary orthopaedic oncology centres in Canada and the UK. Patients who developed multiple extremity STSs were collated retrospectively from prospective oncology databases. A literature review using MEDLINE was also performed. Six patients were identified in the case series from these three institutions, and five studies were identified from the literature review. Overall, 17 patients were identified with a median age of 51 years (range: 19 to 77). The prevalence of this manifestation in STS patients is 1 in 1225. The median disease-free interval between diagnoses was 2.3 years (range: 0 to 19 years). Most patients developed the secondary STS in a metachronous pattern, the remaining, synchronously. The median survival after the first sarcoma was 6 years, and it was 1.6 years after the second sarcoma. The 5-year overall survival rate was 83.3% and 50% following the first and second STS diagnoses, respectively. A diagnosis of two STSs does not confer a worse prognosis than the diagnosis of a single STS. Developing a second STS is a rare event with no identifiable histological pattern of occurrence. Presentation in a metachronous pattern is more common. A high degree of vigilance is required in patients with a previous STS both to detect both local recurrence and to identify new masses remote from the previous STS site. Acquiring an early histological diagnosis should be attempted.

## 1. Introduction

Soft tissue sarcoma (STS) is a general term assigned to a group of rare malignant tumours arising from a mesenchymal stem cell. The term “sarcoma” encompasses approximately 100 different histological subtypes of tumour as per the latest World Health Organization Classification of Tumours of Soft Tissue and Bone (4th edition) [1]. STS accounts for less than 1% of all malignant neoplasms in adults and around 4–8% in children [2]. Prognosis for sarcomas has improved, on average the 5-year overall survival rate being around 60% [3]. However, this figure varies widely depending on the tumour grade, size, stage at diagnosis, depth, location, and histological subtype [4]. The most commonly used staging system for STS (the American Joint Committee on Cancer [5]) uses the first three listed prognostic factors when calculating the stage of a sarcoma.

Commonly, the primary complaint of a patient presenting with a suspected soft tissue sarcoma is the presence of an enlarging, painless mass. Detailed evaluation of the mass and its association with surrounding structures is crucial [6]. Certain types of STSs (synovial sarcoma, epithelioid sarcoma, clear-cell sarcoma, rhabdomyosarcoma, and angiosarcoma) have a tendency to spread via the lymphatic system, thereby warranting palpation of local lymphatics for other masses [7]. However, it is not currently considered routine practice to screen for a second primary soft tissue mass on an initial evaluation for sarcoma.

Although uncommon, patients with STS have an increased risk of developing a second primary neoplasm (carcinoma), even when excluding known syndromic associations such as Li–Fraumeni syndrome and neurofibromatosis [8, 9]. Outside of these syndromes, there have been very few reports on patients developing multiple STSs. Furthermore, there are no reports on how the development of multiple STSs correlates with patient survival. This study aims to elucidate the impact of a diagnosis of multiple STSs on survival and whether there are any associated chronological or histological disease patterns.

## 2. Methods

### 2.1. Patient Identification

Two tertiary sarcoma centres in Canada and one in the United Kingdom collaborated in this study. A retrospective review from 1997 to July 2017 was conducted using the prospectively collated oncology databases at each centre. Electronic notes were searched using a combination of the following terms: “sarcoma,” “multiple,” “new,” “different,” “synchronous,” and “metachronous.” A list of patients was generated when the database notes indicated the possibility of a second primary soft tissue sarcoma. Patients with two or more histologically confirmed extremity STSs were manually identified. Clinical information including the patient demographics, pathologic diagnosis, tumour site, operation, and survival outcomes were gathered from clinical notes. Diagnoses were made by specialized musculoskeletal pathologists at the three centres. No minimum follow-up criteria were assigned in order to maximize inclusion.

In most cases, access to both STS specimens was available to allow direct comparison for accurate histological diagnosis and to distinguish this from a distant soft tissue metastasis of the original primary sarcoma or a radiation-induced sarcoma. Cahan's criteria were used to discern a second primary sarcoma from an iatrogenic sarcoma secondary to radiation, which are a latency period of at least 2 years, the tumour must arise in the direct region of previous radiation, and the sarcoma must be histologically different from the previously radiated lesion [10].

### 2.2. Literature Review

**Figure 1:**
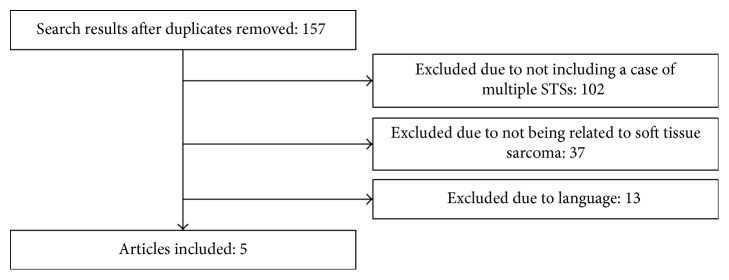
Results of the literature search.

A search was conducted using the MEDLINE OVID interface from 1946 to July 2017. The search strategy used was [Sarcoma OR ST neoplasm] AND [synchronous OR metachronous]. Inclusion criteria included English, human studies with one or more of the subjects with multiple sarcomas. This yielded 157 results, of which five papers were identified that met the inclusion criteria (Figure 1). Manual searching through reference lists was also conducted.

Patients identified in our case series and in the literature review with desmoid tumours, Kaposi sarcoma, and gastrointestinal stromal tumours (GISTs) were excluded from the study due to their unique disease pattern which differs from that of other STSs.

## 3. Results

### 3.1. Case Series

Six patients were identified to meet the study inclusion criteria after detailed interrogation of the databases from the three tertiary orthopaedic oncology centres. Amongst all patients with STS at these centres (*n*=7351), this amounted to a prevalence of 0.08%. The median age of the patients in our series was 54.5 years (range: 28 to 67). Five (83.3%) of the patients were male, and the other was female. The median interval between the diagnosis of the primary and secondary sarcomas was 2.7 years (range: 0.1 to 4.6 years).

Four (66.7%) patients developed sarcomas in a metachronous pattern. The diagnosis interval for the remaining two patients was 1 and 5 months. Although both primary tumours were not diagnosed at the same time, the interval is short enough to be considered a synchronous pattern. Four patients developed their second STS on the ipsilateral side to the first, three of which occurred in different areas of the same limb, while the fourth patient's first STS occurred in the hand followed by one in the lower limb. Liposarcoma was the most common diagnosis, followed by undifferentiated pleomorphic sarcoma (Table 1).

The median time from first sarcoma diagnosis to last follow-up was 7.7 years (range: 0.8 to 12.9). Five (83.3%) patients were alive with no evidence of disease at last follow-up. One patient developed a local recurrence 33 months after resection of their second sarcoma; however, this was resected, and the patient remained free of disease before dying 7 years later with no evidence of sarcoma recurrence. Two patients developed metastasis amenable to complete metastasectomy at 2 and 60 months after the first and second STS excision, respectively. Following metastasectomy, these patients had no evidence of disease at 10.1 and 3.2 years of follow-up.

All patients were managed under the care of a sarcoma multidisciplinary team (MDT), with treatment following international guidelines active at the time. One patient was treated with radiotherapy for both sarcomas, while five patients received radiation for only one of their two sarcomas. Of the patients who underwent radiotherapy for only one sarcoma, two received it for the first sarcoma and three for the second sarcoma. No patients received adjuvant chemotherapy treatment.

Postoperative surveillance comprised clinical examination for evidence of local and regional recurrence with subsequent magnetic resonance imaging (MRI) based on concerning clinical findings and chest radiography with subsequent computerized tomography (CT) to confirm metastasis based on abnormal radiographs. Follow-up occurred at 3-month intervals for the first 2 years, 6-month intervals until 5 years, and annually until 10 years following resection as per international guidelines [11].

### 3.2. Literature Review

Five patient series were identified that included patients with multiple different histologically confirmed STSs [8, 9, 12–14]. Two of the articles were case reports, and three were case series. From these five articles, 17 additional patients were identified. However, seven patients did not meet our inclusion criteria and were therefore excluded. Six patients were excluded from the study by Grobmyer et al. since the diagnoses include GIST or malignant mixed Mullerian tumour. One patient was excluded from the study by Merimsky et al. due to the possibility of the second sarcoma being radiation-induced [8, 9].

### 3.3. Combination of Case Series and Literature Review Cases

Combining data from our current case series and patients selected from the other five series equated to a total of 17 patients with a median age of 51 years (range: 19 to 77) including 9 males and 6 females (two patients' sex were not reported). The median interval between the first and second STS diagnoses was 2.3 years (range: 0 to 19). This included 6 (35.3%) synchronous manifestations of multiple sarcomas and 11 patients (64.7%) that developed distinct tumours in a metachronous pattern. The most common diagnosis was undifferentiated pleomorphic sarcoma (*n*=8, 23.5%) followed by liposarcoma (*n*=6, 17.6%) and leiomyosarcoma (*n*=5, 14.7%). From available data, 7 (53.8%) patients' second tumours arose on the contralateral side and 6 (46.2%) on the ipsilateral side. Overall, tumours presented as high grade (grade 3) in 57.1% of cases, intermediate grade (grade 2) in 28.6% of cases, and low grade (grade 1) in 14.3% of cases (Tables 1 and 2).

The median time from first tumour diagnosis to death or last follow-up was 6 years (range: 0.2 to 26). The 5-year overall survival rate was 83.3%. Median survival time from the second sarcoma diagnosis was 1.6 years (range: 0 to 19) with a 5-year disease-free survival rate of 50%. Overall, six patients were deceased, and 11 were alive with no evidence of disease at last follow-up.

One series which included four patients did not report information on adjuvant therapy [8]. From the available information pertaining to the other 14 patients, adjuvant or neoadjuvant radiotherapy was utilized in 12 patients (85.7%). Three patients were treated with chemotherapy.

## 4. Discussion

A case series of 6 patients and literature review of 11 patients were conducted on multiple primary STSs occurring in individual patients. The aim of the study was to determine the impact developing a second primary STS had on overall survival and whether there are any predictable histological or chronological patterns to disease presentation. We hoped to better enable clinicians to provide a prognosis when faced with these patients as well as to advise on routine STS follow-up. The prognosis in these patients was found to be poor relative to patients with one STS diagnosis, and no predictable patterns of disease presentation were identified.

There are many possible explanations for the development of multiple sarcomas in a single patient. One possibility is that these patients may have a genetic predisposition to malignant mesenchymal disease that is currently not described. Alternatively, general risk factors for malignancy may have been the underlying cause such as immunocompromise or exposure to carcinogens, either environmental or iatrogenic. These factors have been associated with the formation of cancers [15–17], although uncommonly associated with STS. Many other possibilities can be hypothesized but are difficult to identify due to the rarity of the disease.

The median survival time from the first STS diagnosis was 6 years; however, it was only 1.6 years after the second STS diagnosis. The 5-year disease-free survival was 83.3% and 50%, after the first and second STS diagnoses, respectively. The American Cancer Society reported the mean 5-year survival rate of STS as 64% in 2017 [3]. In our review, the survival rate following the first STS diagnosis compares favourably. However, the 5-year survivorship of 50% following the second STS diagnosis is lower relative to the reported mean. This may be due to the morbidity associated with having a second tumour or the complications associated with additional treatment. However, these survival rates may have been negatively affected by a number of confounding factors, such as the short length of follow-up, the second STS diagnosis representing soft tissue metastases of the primary STS rather than a new primary, or because patients in one of the identified studies generally doing poorly [9]. Isolating patients from our case series, there was no evidence of multiple STSs conferring a poorer prognosis, as five of six patients had no evidence of disease at last follow-up with the other patient dying of unrelated causes. Using available data, it is difficult to determine whether the length of the latent period between the STS diagnoses correlates with survival. However, we could anticipate an improved prognosis following the second STS if this tumour arises whilst patients are still being followed-up at a sarcoma centre for their first STS.

The prevalence of a patient with one STS developing a second STS is low, 0.082% or 1 in 1225. Grobmyer et al. were also able to determine the incidence of developing two STSs. They reported that, in a patient previously diagnosed with STS, the incidence of a second primary sarcoma was 1 per 2500 population, or 12.5 times greater than the standard incidence of STS [8]. Therefore, although still a relatively unusual event, the higher incidence in primary sarcoma patients needs to be kept in mind when following up these patients [18, 19].

No identifiable patterns were identified in terms of distinct STS subtypes presenting together in the same patient. The type of sarcoma that arises secondarily appears to be random, independent of the first sarcoma diagnosis. The time used to define synchronous development was 6 months or less between diagnoses. This cutoff time was chosen as it would be too narrow of an interval to develop two individual primaries meaning that the second tumour likely went unrecognized previously. The median time between sarcoma diagnoses was 2.3 years. Approximately one-third of patients developed the second sarcoma in a synchronous pattern, while in two-thirds, the second sarcoma developed metachronously. Therefore, more attention should be paid to identifying a second STS in patients during the postoperative follow-up period. Over recent years, there has been an increase in the use of whole-body staging studies, such as fluorodeoxyglucose positron emission tomography (FDG-PET) or whole body MRI. This may lead to an earlier detection of a second primary tumour in those patients who are undergoing staging at the initial diagnosis but also have a synchronous second primary STS. However, the cost and availability associated with the routine use of these tests need to be considered in light of the rarity of a diagnosis of a second soft tissue sarcoma.

The occurrence of a second STS in the same patient is very rare, and therefore, no change to the management of new patients or patient follow-up is recommended. During follow-up visits for a primary STS, a short screening phrase such as “Have you noticed any new lumps or bumps?” is recommended to raise attention to any newly developing masses. Any second mass a patient develops should not be presumed to be the same disease, and new histological diagnosis should be made. Therefore, we recommend reimaging, restaging, and a biopsy to help diagnose new disease, including radiation-induced tumours, from recurrence or metastasis [18]. In fact, new disease should always be suspected. Merimsky et al. noticed that the risk of developing a secondary neoplasm in patients with STS is significantly greater than that in the general population, 7.5% compared to 1% [9]. Therefore, thorough screening for new STSs or other neoplasms should be considered for patients with new symptoms at follow-up since they are at increased risk.

Unfortunately, several limitations to interpreting these figures exist including the short follow-up period of many patients. Only 12 of the 17 patients had follow-up to five years after diagnosis of their first sarcoma. As well, all patients from one series had very short survival after the second STS diagnosis, which may have skewed the results [9]. Another limitation includes the limited number of patients, hand-selected from observational data potentially introducing selection bias. Although all histologies were reviewed by a specialist musculoskeletal pathologist, they were not reviewed by the same pathologist, which may have slightly altered certain diagnoses. There were six cases identified in the literature review with the same histological diagnosis for the first and second sarcomas. Although reported as different sarcomas, we are uncertain as to whether these cases truly represent multiple distinct STSs or soft tissue metastases of previous sarcomas. Genetic testing was not routinely conducted; however, this could be considered for future studies. Although these limitations are recognized, this study helps to clarify this rare manifestation.

Further research is recommended as current available evidence is limited. Larger series of other orthopaedic oncology centres' experience and more case reports are encouraged to increase the amount of described information. This will hopefully help to uncover any patterns of disease occurrence which may lead to identifying risk factors or certain genetic predispositions.

## 5. Conclusion

In conclusion, this study aimed to help define the pattern of disease and outcomes in patients with nonsyndromic, non-radiation-induced multiple extremity STSs. The incidence of this scenario is very low, and more reports are encouraged. This study was unable to definitively demonstrate that patient prognosis with multiple STSs is poor relative to those diagnosed with a single sarcoma. A high index of suspicion should be maintained to detect a second STS in patients with a current or previous primary STS as they are at an increased risk compared to the general population. Our results demonstrate that majority of patients with multiple STSs present in a metachronous pattern of disease and require routine long-term screening. Management of the second sarcoma should continue in line with internationally recognized guidelines. The recommendations for practice include thorough screening for any new symptoms as well as ensuring a second histological diagnosis is made in the setting of a new mass.

## Figures and Tables

**Table 1:** Case series: patient demographics and outcomes.

Patient	Age	Gender	First tumour type	Location	Second tumour type	Location	Radiotherapy (1st or 2nd)	Time between diagnoses (months)	Diagnosis to last f/u (months)	Status at last f/u
1	28	M	Pseudomyogenic hemangioendothelioma	R 5th metatarsal	Myxoid liposarcoma	R popliteal	Yes (2)	1	6	NED
2	60	M	Pleomorphic liposarcoma	L groin	Epitheliod hemangioendothelioma	L thigh and calf	Yes (1)	28	32	NED
3	67	F	Fibrosarcoma	R forearm	Undifferentiated pleomorphic sarcoma	L buttock	Yes (1)	41	137	NED
4	49	M	Epithelioid sarcoma	L thumb	Myxoid liposarcoma	L popliteal	Yes (2)	5	125	NED
5	67	M	Undifferentiated pleomorphic sarcoma	R calf	Malignant solitary fibrous tumour	R ankle	Yes (2)	55	155	Deceased
6	38	M	Malignant solitary fibrous tumour	L thigh	Undifferentiated pleomorphic sarcoma	R chest wall	Yes (1 + 2)	36	60	NED

M = male; F = female; R = right; L = left; 1 = radiotherapy for the first sarcoma; 2 = radiotherapy for the second sarcoma; NED = no evidence of disease; f/u = follow-up.

**Table 2:** Literature review: patient demographics and outcomes by the study [8, 9, 12–14].

Study	Patient	Age	Gender	First tumour type	Location	Second tumour type	Location	Adjuvant treatment	Time between diagnosis (years)	Diagnosis to last f/u (years)
Schiffman [12]	1	26	M	Synovial sarcoma	L knee	Epithelioid sarcoma	L knee	CTx and RTx	7	26 (DOD)
Merimsky et al. [9]	2	—	—	Meningioma	Frontal lobe	Liposarcoma	Thigh	RTx and CTx	15	16.6 (NED)
	3	—	—	Undifferentiated pleomorphic sarcoma	Thigh	Undifferentiated pleomorphic sarcoma	Buttock	None	19	20.5 (NED)
Grobmyer et al. [8]	4	77	M	Dedifferentiated liposarcoma	Extremity	Solitary fibrous tumour	—	—	0	1 (NED)
	5	51	F	Well-differentiated liposarcoma	Extremity	Leiomyosarcoma	Retroperitoneum	—	0	0.16 (NED)
	6	56	F	Dermatofibrosarcoma protuberans	Extremity	Solitary fibrous tumour	Spine	—	0.5	0.5 (NED)
Daigeler et al. [13]	7	—	F	Leiomyosarcoma	R thigh	Leiomyosarcoma	L thigh	RTx	1.3	1.9 (DOD)
	8	—	M	Undifferentiated pleomorphic sarcoma	L thigh	Undifferentiated pleomorphic sarcoma	R thigh/groin	RTx	0.3	3.1 (DOD)
	9	—	M	Clear-cell sarcoma	R elbow	Clear-cell sarcoma	L elbow	RTx and CTx	9.5	11 (DOD)
	10	—	F	Leiomyosarcoma	L shin	Leiomyosarcoma	R calf	—	5.5	7.5 (DOD)
Scepanovic et al. [14]	11	19	F	Undifferentiated pleomorphic sarcoma	R shoulder	Undifferentiated pleomorphic sarcoma	L shoulder	RTx	1.5	6 (NED)

M = male; F = female; R = right; L = left; CTx = chemotherapy; RTx = radiotherapy; DOD = dead of disease; NED = no evidence of disease; f/u = follow-up.
